# Visualizing Oxysterol-binding Protein Ligand-Triggered Structural Signals

**DOI:** 10.1063/4.0000949

**Published:** 2025-10-27

**Authors:** Walter Galie, Ruth Fiona Bayimenye, Anthony Burgett, Christina Bourne

**Affiliations:** 1 The University of Oklahoma, Norman, OK, USA; 2 The University of Oklahoma Health Sciences, Oklahoma City, OK, USA

## Abstract

Oxysterol-binding protein (OSBP) is involved in targeted lipid transport between cellular organelles, influencing cell signaling, mTOR activation, and other events important for homeostasis. OSBP resides between the Golgi apparatus and the endoplasmic reticulum (ER), transferring phosphatidylinositol 4-phosphate (PI(4)P) to the ER and cholesterol to the Golgi in a retrograde manner using its oxysterol-related domain (ORD). Infections mediated by RNA-based viruses, including Hepatis C, Dengue, Zika, and Coronaviruses, take advantage of OSBP’s lipid transfer function to construct membrane bound viral replication organelles using host-supplied lipids. *In vivo* studies demonstrate that OSBP is a druggable target that blocks viral replication in response to structurally diverse small molecule inhibitors (i.e., OSW-1, itraconazole).^1^

Furthermore, OSBP plays a role in a number of various cancers, driving further investigation into the structure-function relationship to understand it’s involvement in disease states.

We hypothesize that distinct structural changes arise upon binding to different structurally diverse ligands, and that these changes in structure correlate to different cellular localization and functions observed in phenotypic data. Limited structural data for this important family of proteins significantly hampers further chemical biology or medicinal chemistry approaches. The focus of our research includes diverse structural approaches including cryogenic electron microscopy (cryo-EM) and small-angle x-ray scattering (SAXS) for structural determination of the OSBP full-length protein. Synergistically, the ORD is being studied using crystallographic techniques to obtain high-resolution maps of critical contacts between ligand and this region of the protein. Our structural studies will enable determination of critical contacts between OSBP and ligands to assist in developing more potent inhibitors of OSBP, including enhancement of anti-viral effects and potential new binding modes. Overall, this will directly fill gaps in structural relationships to function, positively contributing to the development of new medicines and modalities of anti-viral and anti-cancer treatments.

## Notes

We hypothesize that distinct structural changes arise upon binding to different structurally diverse ligands, and that these changes in structure correlate to different cellular localization and functions observed in phenotypic data. Limited structural data for this important family of proteins significantly hampers further chemical biology or medicinal chemistry approaches. The focus of our research includes diverse structural approaches including cryogenic electron microscopy (cryo-EM) and small-angle x-ray scattering (SAXS) for structural determination of the OSBP full-length protein. Synergistically, the ORD is being studied using crystallographic techniques to obtain high-resolution maps of critical contacts between ligand and this region of the protein. Our structural studies will enable determination of critical contacts between OSBP and ligands to assist in developing more potent inhibitors of OSBP, including enhancement of anti-viral effects and potential new binding modes. Overall, this will directly fill gaps in structural relationships to function, positively contributing to the development of new medicines and modalities of anti-viral and anti-cancer treatments.
